# Hypoxia ameliorates maternal diet‐induced insulin resistance during pregnancy while having a detrimental effect on the placenta

**DOI:** 10.14814/phy2.15302

**Published:** 2022-05-10

**Authors:** Niina Sissala, Elisa Myllymäki, Florian Mohr, Riikka Halmetoja, Paula Kuvaja, Elitsa Y. Dimova, Peppi Koivunen

**Affiliations:** ^1^ Biocenter Oulu and Faculty of Biochemistry and Molecular Medicine Oulu Center for Cell‐Matrix Research University of Oulu Oulu Finland; ^2^ Finnish Institute for Health and Welfare Oulu Finland

**Keywords:** gestational diabetes, glucose tolerance, hypoxia

## Abstract

Maternal overweight/obesity contributes significantly to the development of gestational diabetes, which causes risks to both mother and fetus and is increasing sharply in prevalence worldwide. Since hypoxia reprograms energy metabolism and can alleviate weight gain, adiposity, insulin resistance (IR), and dyslipidemia, we set out to study the potential of sustained reduced ambient oxygen tension (15% O_2_) during pregnancy for alleviating the detrimental effects of diet‐induced IR in C57Bl/6N mice, taking normal chow‐fed and normoxia (21% O_2_) groups as controls. Our data show that hypoxic intervention reduced maternal weight gain, adiposity, and adipose tissue inflammation, and ameliorated maternal glucose metabolism and IR during gestation in diet‐induced IR relative to normoxia. Where diet‐induced IR reduced maternal hemoglobin and increased serum erythropoietin levels, hypoxic intervention compensated for these changes. Diet‐induced IR reduced fetal growth in normoxia, and even more in hypoxia. Hypoxic intervention reduced liver weight gain during pregnancy in the dams with diet‐induced IR, maternal liver weight being positively associated with embryo number. In case of diet‐induced IR, the hypoxic intervention compromised placental energy metabolism and vascularization and increased end‐pregnancy placental necrosis. Altogether, these data show that although hypoxic intervention mediates several beneficial effects on maternal metabolism, the combination of it with diet‐induced IR is even more detrimental to the placental and fetal outcome than diet‐induced IR alone.

## Graphical abstract

In a mouse model, exposure to hypoxic environment for the duration of pregnancy reduced weight gain, improved glucose tolerance, and alleviated insulin resistance of dams with diet‐induced obesity. Placental vascularization, which supports fetal development and growth, was decreased by hypoxic intervention in dams with diet‐induced obesity. Prolonged hypoxia during pregnancy conveys several beneficial effects on maternal metabolism but in combination with diet‐induced obesity is detrimental to the feto‐placental unit and therefore not as such a suitable method for treatment of gestational diabetes.

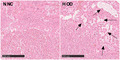

## 1 INTRODUCTION

All aerobic organisms require oxygen for their cellular functions, the majority of this oxygen, >90%, being used for energy production by oxidative phosphorylation (OXPHOS) in mitochondria. Oxygen deficiency, that is hypoxia, is therefore a hazard for which sensing and rescue mechanisms have evolved (Kaelin & Ratcliffe, 2008; Koivunen & Kietzmann, 2018; Koivunen et al., 2016; Semenza, 2012). These include the transcriptional response mediated by the hypoxia‐inducible factor (HIF)/HIF prolyl 4‐hydroxylase (HIF‐P4H) pathway, where the former regulates the expression of hundreds of genes to alleviate hypoxia and the latter acts as a cellular oxygen sensor (Downes et al., 2018; Kaelin & Ratcliffe, 2008; Koivunen & Kietzmann, 2018; Koivunen et al., 2016; Semenza, 2012). The HIF target genes include many of those involved in glucose and lipid metabolism, with the aim of reprogramming energy metabolism to induce non‐oxygen‐demanding glycolysis and glucose intake that is independent of insulin, and of reducing OXPHOS (Kierans & Taylor, 2021; Koivunen et al., 2016). Other key processes mediated by the HIF response are erythropoiesis and angiogenesis, where the well‐established HIF targets erythropoietin (EPO) and vascular endothelial growth factor (VEGF) increases oxygen availability and delivery (Haase, 2013). Small molecule HIF‐P4H inhibitors, which activate the HIF response under normoxia, have recently been accepted for the treatment of anemia in cases of chronic kidney disease (Dhillon, 2019). Clinical data from anemia patients have indicated that, in addition to heightened hemoglobin (Hb) levels, HIF‐P4H inhibitors lead to lower serum cholesterol and triglyceride levels (Flamme et al., 2014; Olson et al., 2014; Provenzano et al., 2016). Activation of the HIF response in mice through a genetic deficiency in the most abundant isoenzyme and the main one regulating HIF, namely HIF‐P4H‐2, has shown protection against obesity, metabolic dysfunction, insulin resistance (IR), fatty liver disease, and atherosclerosis, suggesting that activation of the HIF response is a potent means of regulating metabolism (Laitakari et al., 2020; Rahtu‐Korpela et al., 2014, 2016).

Overweight and obesity promote gestational diabetes mellitus (GDM), which has become an increasingly common condition complicating pregnancies, with a global prevalence of 2%–25%, varying between countries (McIntyre et al., 2019; Szmuilowicz et al., 2019; The Finnish Institute for Health (THL), 2020). GDM poses significant short‐term and long‐term adverse health problems for both mother and child (Damm et al., 2016). The short‐term risks associated with GDM include increased rates of gestational hypertension, pre‐eclampsia and Caesarean section for the mother, and macrosomia and perinatal hypoglycemia for the fetus (Vääräsmäki, 2016). In the longer term, both women with a history of GDM and their offspring have an increased risk of metabolic and vascular diseases (Damm et al., 2016; Vääräsmäki, 2016; Zhang et al., 2016). Diet and lifestyle interventions are the first line treatment options for GDM, while therapeutic treatment for hyperglycemia can be started if these are not sufficient (Vääräsmäki, 2016).

It is known that being exposed to hypoxia by living at a high altitude can reduce birth weight by 100 g/1000 m, an effect that is associated with increased infant mortality and morbidity (Jensen & Moore, 1997). A large number of rodent studies have demonstrated that maternal hypoxia during pregnancy, caused either by maternal inhalation hypoxia or reduced uterine perfusion pressure, affects maternal metabolism and is particularly harmful to the placenta and the developing fetus (Siragher & Sferruzzi‐Perri, 2021). In case of maternal inhalation hypoxia, intensity, timing, and duration appear to be important for determining the effects; a change of phenotype occurring around 12% O_2_ below which the effects are more detrimental on placenta and fetus (Siragher & Sferruzzi‐Perri, 2021). The reported alterations of hypoxia to maternal metabolism in rodent studies have been reduced maternal adiposity, altered glucose and lipid metabolism, and altered concentrations of circulating hormones and inflammatory markers (Siragher & Sferruzzi‐Perri, 2021). We have shown earlier in normal chow (NC)‐fed C57Bl/6N dams that exposure continuous moderate ambient normobaric hypoxia (15% O_2_, which equals altitude of 2700 m) during gestation (E0.5 onwards) reduced fetal weight by 7% at the end of gestation (E17.5) relative to normoxia (21% O_2_) while at the same time significantly modifying the maternal metabolism (Määttä et al., 2018). The reduction in fetal weight was clearly less than those reported in severe hypoxia (≤12%) ranging to 39% reduction (Siragher & Sferruzzi‐Perri, 2021). The amount of gonadal white adipose tissue (WAT), which correlated positively with birth weight, was lower in the continuous moderate hypoxia dams (Määttä et al., 2018). The continuous moderate hypoxia dams also had better glucose tolerance at mid‐gestation (E9.5) and end‐gestation than the normoxia dams and failed to develop the same level of IR during late gestation as in the normoxia group, this being associated with a higher glucose intake in the maternal tissues and lower availability of glucose to support fetal growth (Määttä et al., 2018). These metabolic changes were associated with the upregulation of the glycolytic HIF response in maternal tissues (Määttä et al., 2018). Opposite to these findings, severe hypoxia at late gestation has been reported to increase maternal serum corticosterone and glucose levels (Cuffe et al., 2014).

Despite the fetal growth‐hindering effects of hypoxia in the metabolically healthy dams, we hypothesized that exposure to continuous moderate ambient hypoxia (15% O_2_) during pregnancy might alleviate the detrimental metabolic effects and fetal macrosomia observed in dams with diet‐induced IR, and set out to test this using NC‐fed normoxia dams as controls, also assessing the safety of such an intervention.

## 2 MATERIALS AND METHODS

### 2.1 Ethical approval

All the experiments were conducted according to the Finnish Act on Animal Experimentation (62/2006) and approved by the National Animal Experiment Board of Finland (license number ESAVI/8179) and the study is reported in accordance with ARRIVE guidelines.

### 2.2 Animal experiments

2‐month‐old female C57Bl/6NCrl littermates were randomly assigned to one of two diets fed *ad libitum* throughout the experiment: Either (1) an energy‐rich, highly palatable obesogenic diet (OD) (20% fat, 28% polysaccharide, 10% simple sugars, 23% protein [w/w] fortified with AIN‐93‐VX and AIN‐93 M‐MX, Special Diets Services, Witham, UK) supplemented with sweetened condensed milk (8% fat, 57% carbohydrates, 7% proteins [w/w], Dovgan, The Netherlands) to induce IR and weight gain, or (2) NC (6.2% fat, 44.2% carbohydrate, 18.6% protein [w/w], Harlan Teklad, USA). After 3.5 weeks on the respective diets, the development of IR was verified by taking blood samples for the analysis of glucose and insulin levels and calculation of a homeostatic model assessment of insulin resistance (HOMA‐IR) scores from the fasted glucose and insulin values ((fasting insulin (pmol/l) × fasting glucose (mmol/l))/156.65).

After 5 weeks on the respective diets, the mice were mated under normoxic conditions (21% O_2_) overnight and divided the next morning (E0.5) into four groups: normoxia NC (N NC), hypoxia NC (H NC), normoxia OD (N OD) and hypoxia OD (H OD). The mice receiving each diet were assigned to the normoxia and hypoxia groups on the basis of their average body weight and averaged HOMA‐IR. The mice in the hypoxia groups were placed into a hypoxic chamber (Hypoxic Glove Box, Coy Laboratory Products, USA) under a 15% normobaric oxygen concentration (corresponding to an oxygen tension of 2700 m) and those in the normoxia groups were kept in the same room under the equivalent normoxic conditions. The dams were weighed 5 days a week.

The dams fasted for 4 h before sacrifice using CO_2_ and the embryos were sacrificed by decapitation at E9.5 or E17.5. Terminal blood and tissues were collected and maternal Hb and glucose concentrations were measured from the terminal blood, and serum fractions set aside. Gonadal WAT, liver, and placentas were weighed, and tissues were snap frozen in liquid nitrogen. The number of embryos was counted and the embryos and placentas were stripped of fetal membranes and pooled for measurement of the embryonic and placental weight for each dam.

### 2.3 Glucose tolerance test (GTT) and determination of serum insulin and glucagon levels

GTT was performed on the dams after a 4 h fast at E17.5. The mice were anesthetized with fentanyl and midazolam and injected intraperitoneally with glucose (2 mg/g body weight). Blood glucose concentrations were monitored with a glucometer. Serum insulin and glucagon values were determined enzymatically with a Rat/Mouse Insulin ELISA kit (EZRMI‐13K, Millipore) on E9.5 experiment, Ultra Sensitive Mouse Insulin ELISA kit (90080, CrystalChem) on E17.5 experiment and with Mouse Glucagon ELISA kit (81518, CrystalChem) on E17.5 experiment. HOMA‐IR scores were calculated from the fasting glucose and insulin values.

### 2.4 Determination of blood parameters

Blood Hb was measured spectrophotometrically with a HemoCue Hb 201 Analyzer. Total cholesterol, HDL cholesterol, and triglyceride levels were measured from the serum fraction by an enzymatic method (Roche Diagnostics). Fatty acids were determined with a fluorometric Free Fatty Acid Quantification Kit (ab65341, Abcam) and ketoacids with a β‐Hydroxybutyrate Assay kit (MAK041, Sigma Aldrich). Erythropoietin was determined with a Mouse Erythropoietin/EPO Quantikine ELISA kit (MEP00B, R&D Systems).

### 2.5 Histological analyses

Formalin‐fixed paraffin‐embedded placenta and WAT samples were cut to 5 µm sections and stained with hematoxylin‐eosin (HE) or Masson’s Trichrome. Whole‐slide images were scanned with a Hamamatsu NanoZoomer S60 slide scanner. The gonadal WAT adipocyte area was measured in five representative parts of the HE stained whole‐slide images, and the 50 largest adipocytes within the five selected spots were measured with QuPath 3.0 at 20x magnification. Placental layer areas were measured with QuPath 3.0 from the Masson’s Trichrome‐stained whole‐slide images. The >0.5 mm blood vessel cross‐sections in the junctional zone on the HE‐ined placental slides were quantified by an experienced pathologist (P. Ku.) using an Olympus BX41 microscope at 40x magnification.

### 2.6 Quantitative real‐time PCR (qPCR) analyses

Total RNA from the tissues was isolated with the E.Z.N.A. total RNA Kit II (Omega Bio‐Tek) or TriPure Isolation Reagent (Roche Applied Science) and purified with the E.Z.N.A. total RNA Kit I (Omega Bio‐Tek). Reverse transcription was performed with the qScript cDNA Synthesis Kit (Quanta Biosciences). qPCR was carried out using iTaq SYBR Green Supermix with ROX (Bio‐Rad) in a C1000 Touch Thermal Cycler and CFX96 Touch Real‐Time PCR Detection System (Bio‐Rad) with the primers shown in Table 1. Actin beta (*Actb*) was used as a reference gene for skeletal muscle, kidney and gonadal WAT, TATA box binding protein (*Tbp*) for placenta and the geometrical mean of beta‐2 microglobulin (*B2m*) and *Tbp* for liver.

**TABLE 1:** Sequences of the primers used in qPCR analysis

Gene	Forward primer	Reverse primer
*Actb*	AGAGGGAAATCGTGCGTGAC	CAATAGTGATGACCTGGCCGT
*B2m*	GGTCTTTCTGGTGCTTGTCTCA	GTTCGGCTTCCCATTCTCC
*Tbp*	AGAACAATCCAGACTAGCAGCA	GGGAACTTCACATCACAGCTC
*Gbe1*	ACTGCTTTGATGGCTTCCGT	AACCTTGAACCCATTCCGTGG
*G6pc*	CGACTCGCTATCTCCAAGTGA	GTTGAACCAGTCTCCGACCA
*Gyg*	GCTGGTCACTTACTCAGTATTCC	AGGGTTGATAGACAAAGACTCCA
*Glut1*	TCAAACATGGAACCACCGCTA	AAGAGGCCGACAGAGAAGGAA
*Glut3*	ATGGGGACAACGAAGGTGAC	GTCTCAGGTGCATTGATGACTC
*Eno1*	TGCGTCCACTGGCATCTAC	CAGAGCAGGCGCAATAGTTTTA
*Irs2*	GTAGTTCAGGTCGCCTCTGC	TTGGGACCACCACTCCTAAG
*Ldha*	GCATGAGCTTGCCCTTGTTGA	GACCAGCTTGGAGTTCGCAGTTA
*Pgk1*	GGAGCGGGTCGTGATGA	GCCTTGATCCTTTGGTTGTTTG
*Pdk1*	AGGATCAGAAACCGGCACAAT	GTGCTGGTTGAGTAGCATTCTAA
*Pfkl*	TGCAGCCTACAATCTGCTCC	GTCAAGTGTGCGTAGTTCTGA
*Pparg*	GCCCACCAACTTCGGAATC	TGCGAGTGGTCTTCCATCAC
*Srebp1c*	GAGCCATGGATTGCACATTT	CTCAGGAGAGTTGGCACCTG
*Acaca*	GAAGTCAGAGCCACGGCACA	GGCAATCTCAGTTCAAGCCAGTC
*Fasn*	TCCTGGAACGAGAACACGATCT	GAGACGTGTCACTCCTGGACTTG
*Scd1*	TTCTTGCGATACACTCTGGTGC	CGGGATTGAATGTTCTTGTCGT
*Angpt2*	GAAGCCTGAGAATACCAACCG	CCTTGCTTATAGGTCTCCCAGT
*Angpt4*	AGATCCAGCAATTGTTCCAGAAG	AAGAGGTCTATCTGGCTCTGAAGATT
*Tie1*	CAAGGTCACACACACGGTGAA	GCCAGTCTAGGGTATTGAAGTACG
*vWF*	CTTCTGTACGCCTCAGCTATG	GCCGTTGTAATTCCCACACAAG
*Vegfa*	GCACTGGACCCTGGCTTTAC	AACTTGATCACTTCATGGGACTTCT
*Bcl2*	CTCGTCGCTACCGTCGTGACTTCG	CAGATGCCGGTTCAGGTACTCAGTC
*Caspase3*	ATGGAGAACAACAAAACCTCAGT	TTGCTCCCATGTATGGTCTTTAC

### 2.7 Western blot analyses

Total protein lysates from placentas were extracted with a buffer containing 50 mM TRIS‐HCl, pH 8, 150 mM NaCl, 0.5% NP‐40 and Pierce Protease and Phosphatase Inhibitor Mini Tablets (ThermoScientific). Protein content was measured with the Bradford assay (Bio‐Rad). 100 µg of total proteins were resolved by SDS‐PAGE, blotted, and probed with primary antibodies against Caspase 3 (9662S, Cell Signaling), Cleaved Caspase‐3 (D175) (9661S, Cell Signaling), and PECAM‐1 (CD31) (M20) (sc‐1506‐R, Santa Cruz Biotechnology) followed by HRP‐conjugated secondary antibody (1:5000, Dako). A β‐actin antibody (AC‐15) (NB600‐501, Novus Biologicals) was used as the loading control. Clarity Max Western ECL substrate (Bio‐Rad) was used for detection. The densities of the bands were quantified with Fiji (ImageJ) and normalized to β‐actin.

### 2.8 Determination of hepatic, placental, and WAT triglycerides

Hepatic, placental, and gonadal WAT lipids were extracted by digestion overnight in an ethanol‐KOH solution at +55°C followed by centrifugation. The lipids in the supernatant were then precipitated on ice with MgCl_2_. After another centrifugation, the supernatant was assayed for triglycerides by an enzymatic method (Roche Diagnostics) and the absorbance of the colorimetric products was determined with the Infinite M1000 Pro Multimode Plate Reader (Tecan).

### 2.9 Determination of tissue glycogen

After a 4 h fast the mice were sacrificed with CO_2_ and 150 mg tissue samples were collected from the superior lobe of the liver, the placenta, kidney, and skeletal muscle (*M*. *quadriceps femoris*). These samples were snap frozen in liquid nitrogen and stored in −80°C before analysis with a fluorometric Glycogen Assay Kit (700480, Cayman Chemical).

### 2.10 Statistical analyses

Two‐way ANOVA was used to compare statistical significances between four groups, interactions between N NC and H OD or H NC and N OD are not reported. Student’s *t*‐test was used to compare statistical significances between two groups. The area under the curve (AUC) was calculated by a summary measures method. Pearson’s correlation coefficient was used to compare linear dependences. Values of ±3SD were omitted from the statistical analysis. All data are presented as means ± standard deviation (SD). *p* <0.05 were considered statistically significant. Statistical significance in two‐way ANOVA is presented with * and with # in Student’s *t*‐test. The statistical analysis was performed using GraphPad Prism 9.2.0.

## 3 RESULTS

### 3.1 Hypoxic intervention reduced maternal weight gain, adiposity and liver weight gain during gestation in diet‐induced IR

**FIGURE 1:**
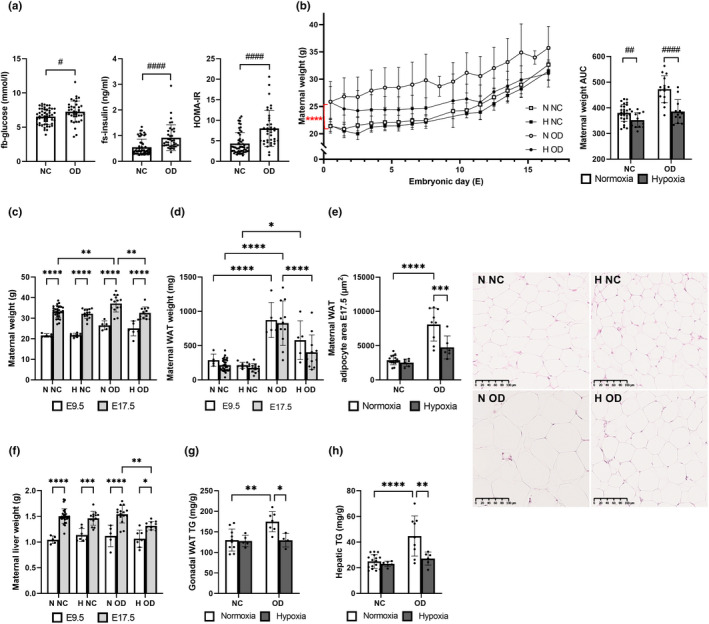
Hypoxic intervention reduced maternal adiposity and liver weight during gestation in diet‐induced insulin resistance. Mice were fed either normal chow (NC) or an obesogenic diet (OD) to induce insulin resistance. During gestation they were housed either in normoxic (N, O_2_ = 21%) or hypoxic (H, O_2_ = 15%) conditions. (a) fb‐glucose, fs‐insulin and HOMA‐IR of non‐pregnant females after 3.5 weeks on the diet (baseline), before hypoxic intervention (NC *n* = 52, OD *n* = 38). (b) Maternal weight gain during gestation and area under the curve (AUC). (c) Maternal weight at sacrifice at E9.5 and E17.5 (E9.5: N NC *n* = 5, H NC *n* = 7, N OD *n* = 5, H OD *n* = 5) (E17.5: N NC *n* = 28, H NC *n* = 13, N OD *n* = 15, H OD *n* = 12). (d) Maternal gonadal WAT weight at E9.5 and E17.5 (E9.5: N NC *n* = 5, H NC *n* = 7, N OD *n* = 5, H OD *n* = 5) (E17.5: N NC *n* = 28, H NC *n* = 13, N OD *n* = 15, H OD *n* = 12). (e) Maternal adipocyte area at E17.5 and corresponding hematoxylin and eosin‐stained histological sections, scale bar 100 µm. (N NC *n* = 16, H NC *n* = 6, N OD *n* = 9, H OD *n* = 6). (f) Maternal liver weight at E9.5 and E17.5 (E9.5: N NC *n* = 5, H NC *n* = 7, N OD *n* = 5, H OD *n* = 5) (E17.5: N NC *n* = 28, H NC *n* = 13, N OD *n* = 15, H OD *n* = 11). (g) Gonadal WAT triglyceride levels at E17.5 (N NC *n* = 19, H NC *n* = 5, N OD *n* = 7, H OD *n* = 4). (h) Hepatic triglyceride levels at E17.5. All data are means ±SD. Two‐way ANOVA *p < 0.05, **p < 0.01, ***p < 0.001, ****p < 0.0001. Student’s t‐test #*p* < 0.05, ##*p *< 0.01, ###*p* < 0.001, ####*p* < 0.0001

The use of an OD to induce pre‐gestation IR in C57BL6/N female mice increased fasting blood glucose levels by >10% (7.3 vs. 6.5 mmol/l), fasting serum insulin levels by ~67% (0.92 vs. 0.55 ng/ml) and HOMA‐IR scores by >85% (8.0 vs. 4.3) by comparison with NC, and also induced a >20% weight gain (25.8 g vs. 21.4 g) during the pre‐gestation period (Figure 1a,b, S1). The insulin‐resistant OD‐fed females and the NC‐fed controls were then mated with NC‐fed males and the pregnant mice were placed either in a hypoxic (15% O_2_ in a normobaric hypoxia chamber) or normoxic environment (21%) for gestation. The mice were sacrificed either at mid‐gestation (E9.5) or end‐gestation (E17.5).

Hypoxic intervention reduced the maternal weight gain during gestation relative to normoxia in the case of both diets, the AUC for maternal weight gain being ~7% less for the group on NC and ~18% less for that on OD and the weight upon sacrifice at E17.5 being ~4% less on NC (*p* = 0.23) and ~10% less on OD (Figure 1b,c). The OD‐fed dams had significantly more WAT and a larger adipocyte area than their NC‐fed counterparts, but the hypoxic intervention reduced these differences, to the extent that the only statistically significant difference at E17.5 was that the hypoxia OD‐fed dams had ~40% smaller adipocytes than the normoxia OD‐fed dams (Figure 1d,e). The combined effect of the hypoxic intervention and diet‐induced IR may be said to have challenged the pregnancy‐induced liver weight gain, leading to lower liver weight at end‐gestation in the hypoxia OD‐fed dams than in their normoxic counterparts (Figure 1f). OD increased gonadal WAT and hepatic triglyceride levels relative to NC in the normoxia dams, while the hypoxic intervention leveled this difference out (Figure 1g,h).

**FIGURE 2:**
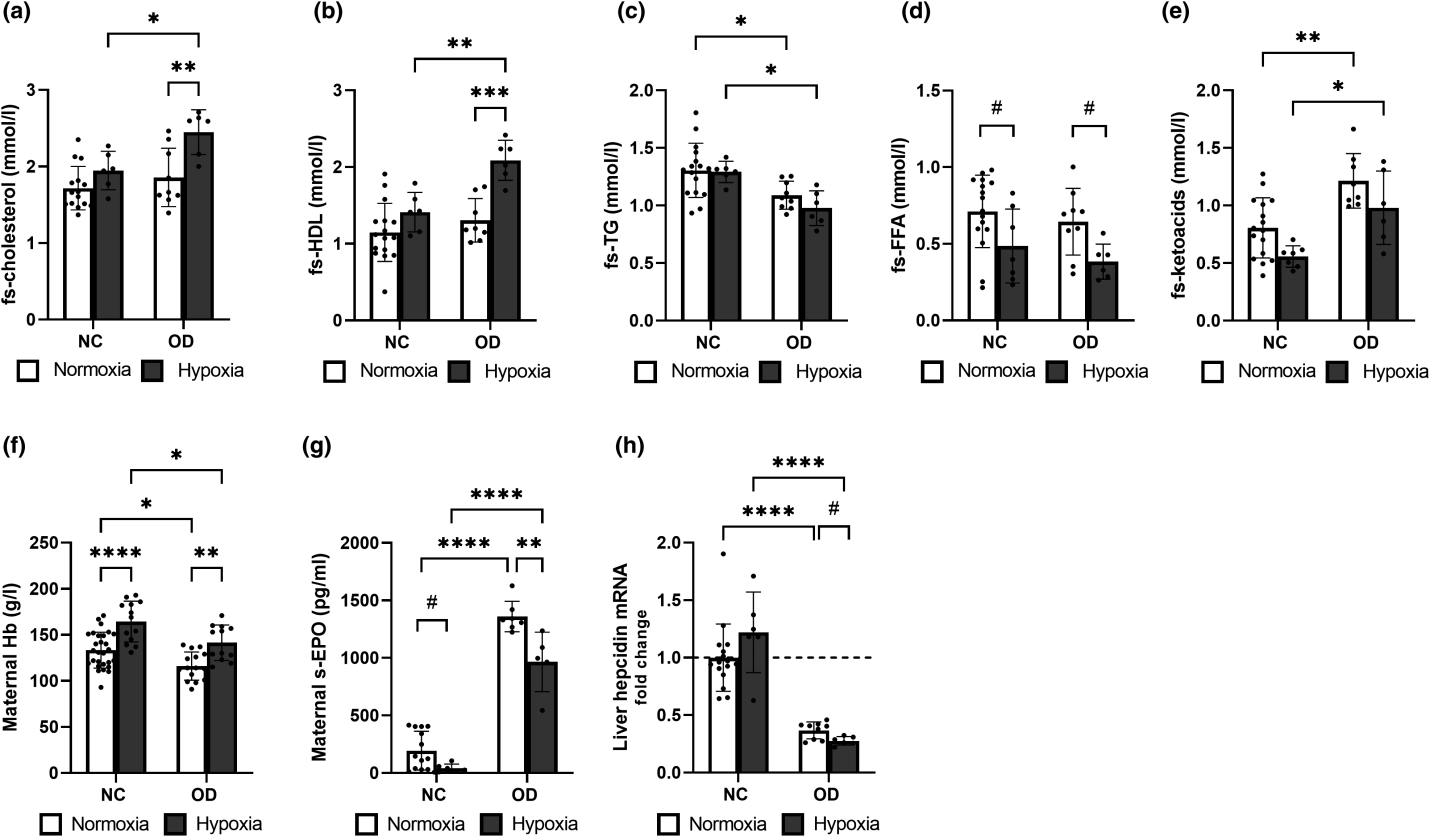
Hypoxic intervention altered serum lipid levels in maternal diet‐induced insulin resistance and changed erythropoietic parameters regardless of the diet at end‐gestation. Mice were fed either normal chow (NC) or an obesogenic diet (OD) to induce insulin resistance. During gestation they were housed either in normoxic (N, O_2_ = 21%) or hypoxic (H, O_2_ = 15%) conditions. The parameters were studied at E17.5. (a) Fasting serum (fs) total cholesterol levels, (b) fs‐HDL cholesterol levels, (c) fs‐triglyceride (TG) levels, (d) fs‐free fatty acid (FFA) levels and (e) fs‐β‐hydroxybutyrate levels. A‐E N NC *n* = 16, H NC *n* = 6, N OD =9, H OD *n* = 6. (f) Maternal hemoglobin (Hb) levels (N NC *n* = 28, H NC *n* = 13, N OD *n* = 15, H OD *n* = 12), (g) maternal serum erythropoietin (EPO) levels (N NC *n* = 14, H NC *n* = 6, N OD *n* = 7, H OD *n* = 5) and (h) maternal liver hepcidin mRNA levels (N NC *n* = 16, H NC *n* = 6, N OD *n* = 9, H OD *n* = 6). All data are mean ± SD. Two‐way ANOVA *p < 0.05, **p < 0.01, ***p < 0.001, ****p < 0.0001. Student’s *t*‐test #*p *< 0.05

The OD increased fasting serum total cholesterol and HDL cholesterol levels at E17.5 in hypoxia but not in normoxia, whereas it reduced fasting serum triglyceride levels relative to NC independent of normoxia or hypoxia (Figure 2a–c). Hypoxia reduced fasting serum FFA levels at E17.5 by >30% in the mice receiving NC and OD as compared with normoxia, with no significant difference between the diets (Figure 2d). The OD increased fasting serum ketoacid levels compared to NC in normoxia and hypoxia at E17.5, with the hypoxic intervention showing a tendency for lower levels than in normoxia in the case of both diets (Figure 2e).

### 3.2 Diet‐induced IR reduced maternal Hb levels and increased serum EPO levels, but hypoxic intervention compensated for these changes

Hypoxic intervention increased maternal Hb levels independent of diet, in agreement with the notion of activation of the hypoxia response being erythropoietic (Figure 2f). The OD reduced maternal Hb levels by ~13% relative to NC in both normoxic and hypoxic environments (Figure 2f), while serum EPO levels were significantly higher in the mice receiving an OD diet than in those on NC, whereas the hypoxic intervention lowered these levels, which was in agreement with the higher Hb levels (Figure 2g). Hepatic hepcidin mRNA levels were downregulated by the OD, and the hypoxic intervention lowered them still further (Figure 2h).

### 3.3 Maternal diet‐induced IR reduced fetal growth in normoxia, and even more in hypoxia

**FIGURE 3:**
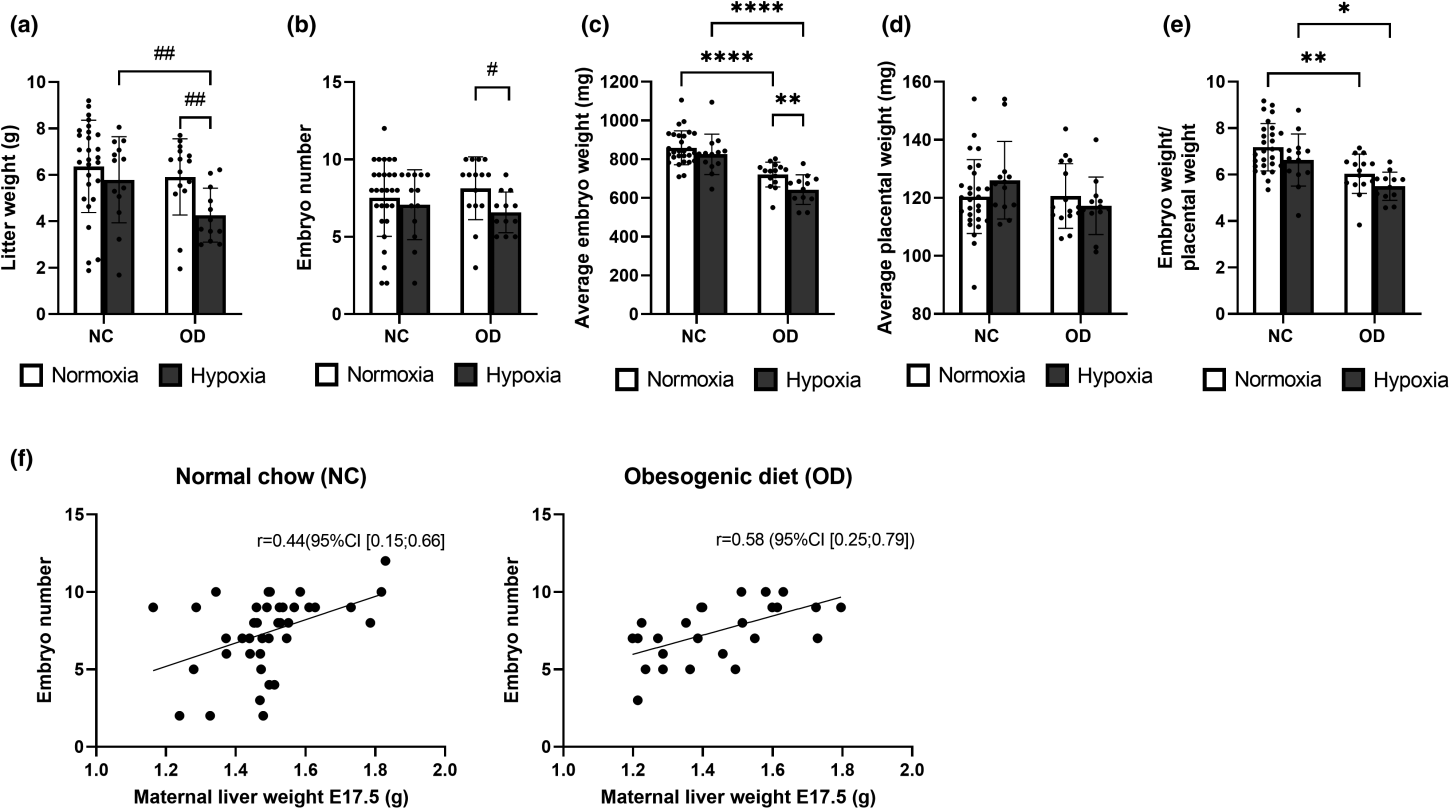
Maternal diet‐induced insulin resistance challenged fetal development and growth in normoxia and even more in hypoxia. Mice were fed either normal chow (NC) or an obesogenic diet (OD) to induce insulin resistance. During gestation they were housed either in normoxic (N, O_2_ = 21%) or hypoxic (H, O_2_ = 15%) conditions. The parameters were studied at E17.5. (a) Litter weight, (b) number of embryos, (c) embryo weight at E17.5, (d) placental weight and (e) ratio of embryo weight to placental weight at E 17.5. A‐D N NC *n* = 28, H NC *n* = 13, N OD *n* = 15, H OD *n* = 12. (f) Correlations between maternal liver weight and the number of embryos when receiving normal chow or an obesogenic diet. All data are means ± SD. Two‐way ANOVA *p < 0.05, **p < 0.01, ***p < 0.001, ****p < 0.0001. Student’s t‐test #*p* < 0.05, ##*p* < 0.01

Litter weight was decreased in the hypoxia OD‐fed dams compared to the normoxia OD‐fed dams and the hypoxia NC‐fed dams (Figure 3a). Although the OD did not have any effect on the number of embryos at E17.5 in normoxia, the combined effect of maternal diet‐induced IR and hypoxia was detrimental to fetal development, reducing the number of embryos in the hypoxic OD‐fed dams by ~20% relative to the normoxic OD‐fed dams (Figure 3b). The OD reduced the weight of the embryos and the embryo weight/placental weight ratio significantly regardless of the normoxic/hypoxic environment, whereas no statistically significant differences were seen in placental weight at E17.5 (Figure 3c–e). Hypoxia reduced the weight of the embryos, the average for the dams receiving the NC diet being ~4% (*p *= 0.38) and that for the OD diet ~10% less than in normoxia, and the embryo weight/placental weight being ~8% (*p *= 0.13) and ~9% (*p *= 0.08) less, respectively (Figure 3c,e). Interestingly, there was a significant positive correlation between the number of embryos and the maternal liver weight in the case of both diets (Figure 3f).

### 3.4 Hypoxic intervention ameliorated maternal glucose metabolism in cases of maternal diet‐induced IR

**FIGURE 4:**
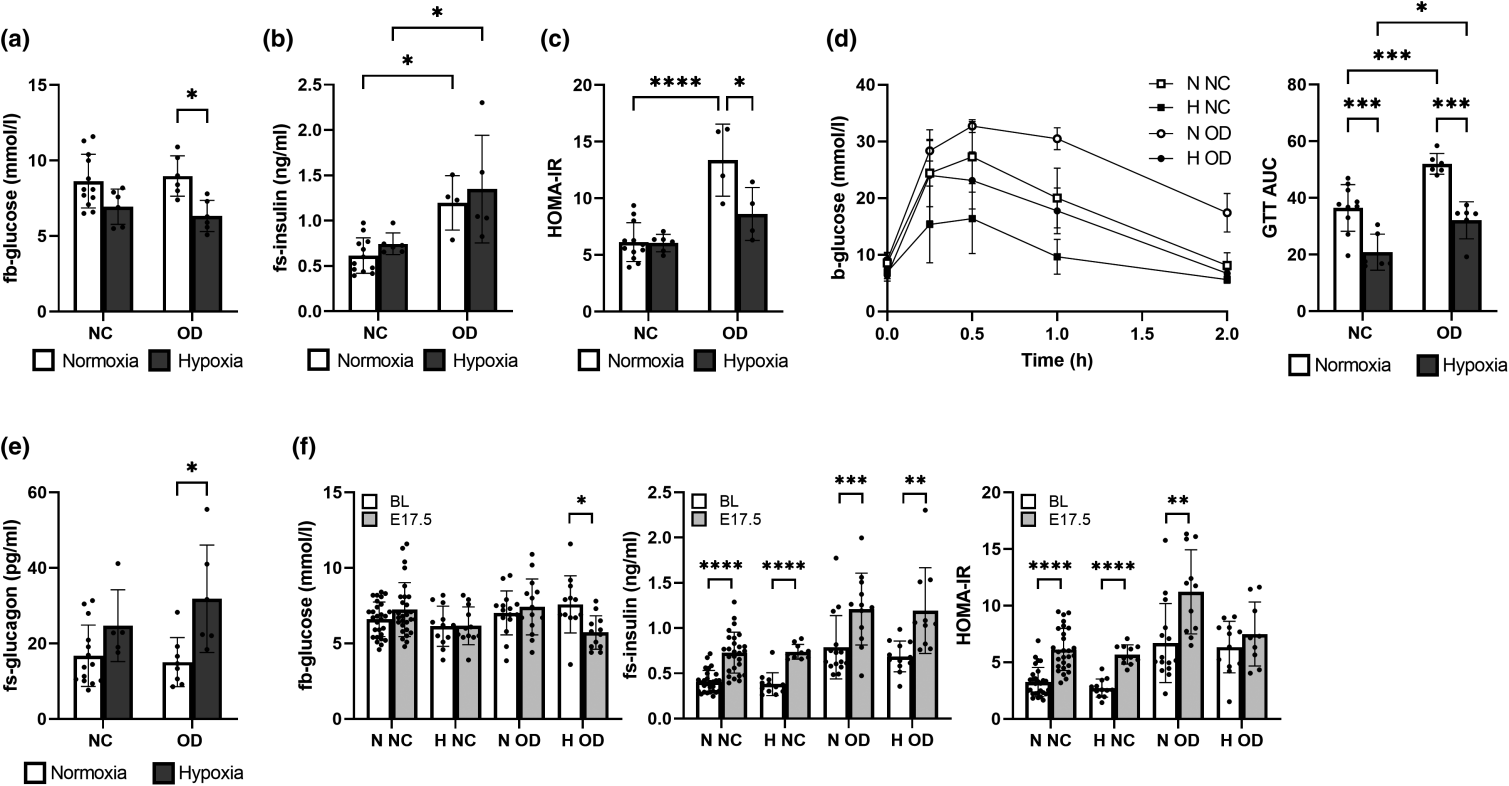
Hypoxic intervention ameliorated glucose metabolism in diet‐induced insulin resistant dams. Mice were fed either normal chow (NC) or an obesogenic diet (OD) to induce insulin resistance. During gestation they were housed either in normoxic (N, O_2_ = 21%) or hypoxic (H, O_2_ = 15%) conditions. Pregnant dams fasted for 4 h before measurement of the parameters. A‐D were measured at E17.5. (a) Fasting blood (fb) glucose levels (N NC *n* = 12, H NC *n* = 6, N OD *n* = 6, H OD *n* = 6). (b) Fasting serum (fs) insulin levels (N NC *n* = 12, H NC *n* = 6, N OD *n* = 4, H OD *n* = 5). (c) HOMA‐IR score (N NC *n* = 12, H NC *n* = 6, N OD *n* = 4, H OD *n* = 5). (d) Glucose tolerance test (GTT) and area under the curve (AUC) (N NC *n* = 10, H NC *n* = 6, N OD *n* = 6, H OD *n* = 6). (e) fs‐glucagon levels (N NC *n* = 14, H NC *n* = 5, N OD *n* = 9, H OD *n* = 6). (f) Changes in fb‐glucose, fs‐insulin, and HOMA‐IR scores between the baseline measurement (pre‐gestation, 3.5 weeks on the diet) and end‐gestation. All data are means ± SD. Two‐way ANOVA *p < 0.05, **p < 0.01, ***p < 0.001, ****p < 0.0001.

Hypoxic intervention reduced fasting blood glucose levels at end‐gestation by 20% in the dams receiving the NC diet (*p *= 0.051) and by 30% in those on the OD (Figure 4a), while the OD increased fasting serum insulin levels relative to NC (Figure 4b). Altogether, these changes contributed to significantly higher HOMA‐IR scores in the normoxia OD‐fed dams than in their normoxia NC‐fed counterparts, and the significantly lower HOMA‐IR scores in the hypoxia OD‐fed dams than in the normoxia OD‐fed dams (Figure 4c). Although the OD worsened glucose tolerance in a GTT relative to NC in both normoxia and hypoxia, the hypoxic intervention improved the situation regardless of the diet, equaling out the difference in outcome between the hypoxia OD‐fed dams and the normoxia NC‐fed dams (Figure 4d). As expected, fasting serum glucagon levels inversely reflected the changes seen in fasting insulin levels, being higher in the hypoxic intervention dams (Figure 4e). Apart from the decline observed in the hypoxia OD‐fed dams, fasting blood glucose levels did not change from the baseline to end‐gestation (Figure 4f). The pregnancy‐induced increase in fasting serum insulin levels was seen at end‐gestation in all the dams (Figure 4a). HOMA‐IR scores almost doubled in the normoxia NC and OD‐fed dams and the hypoxia NC‐fed dams, whereas in the hypoxia OD‐fed dams the combined intervention hindered the rise in the HOMA‐IR scores (Figure 4f).

### 3.5 Diet‐induced IR altered glycogen and glucose metabolism in maternal tissues

**FIGURE 5:**
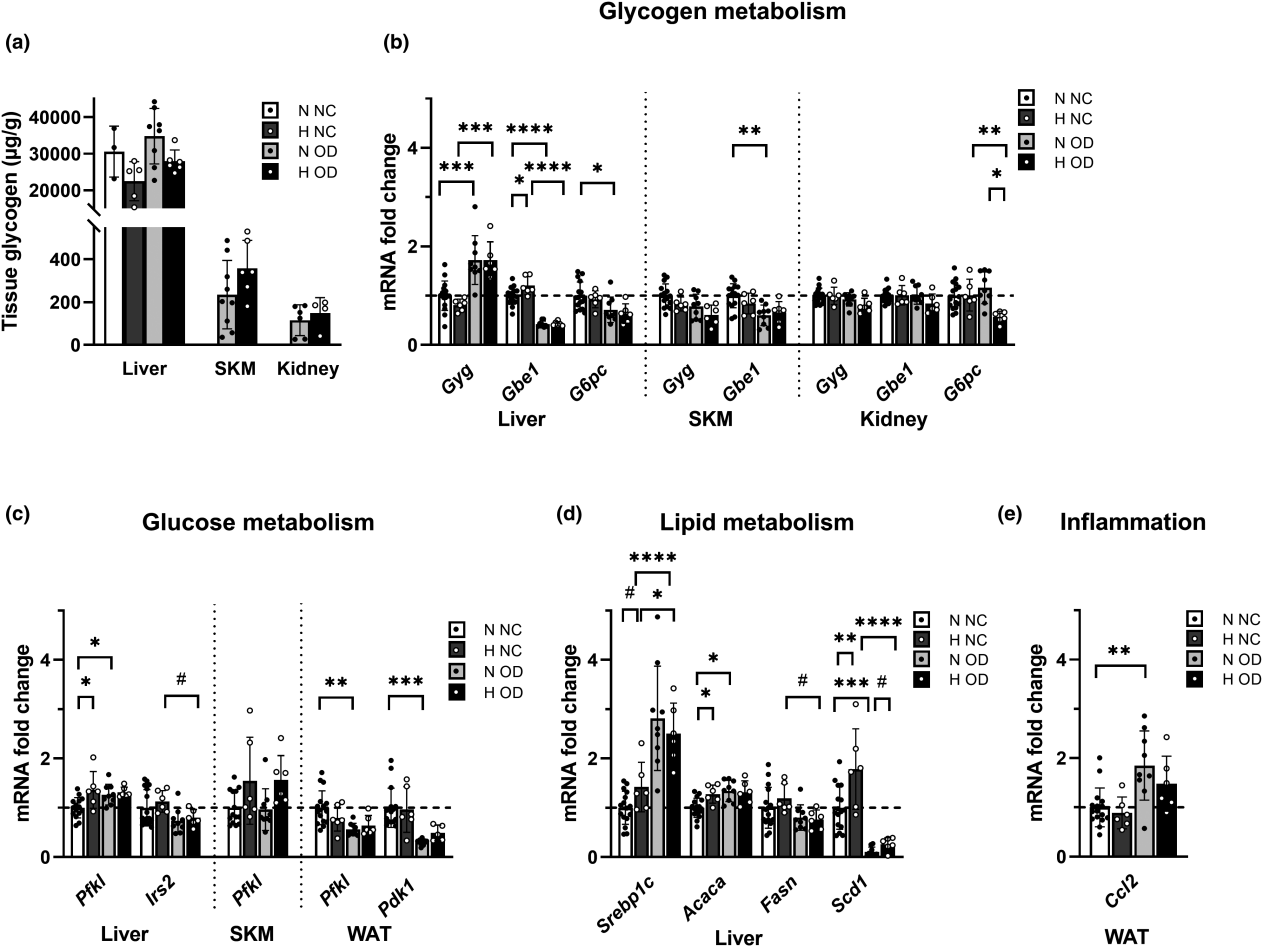
Analysis of maternal tissue glycogen levels and transcript levels of genes involved in glycogen, glucose and lipid metabolism and inflammation. Mice were fed either normal chow (NC) or an obesogenic diet (OD) to induce insulin resistance. During gestation they were housed either in normoxic (N, O_2_ = 21%) or hypoxic (H, O_2_ = 15%) conditions. The parameters were measured at E17.5. (a) Maternal liver glycogen (N NC *n* = 3, H NC *n* = 5, N OD *n* = 8, H OD *n* = 6), maternal skeletal muscle glycogen (N OD *n* = 9, H OD *n* = 6), maternal kidney glycogen (N OD *n* = 9, H OD *n* = 9). (b) Transcript levels of glycogen metabolism genes in liver, skeletal muscle and kidney. (c) Transcript levels of glucose metabolism genes in liver, skeletal muscle and gonadal WAT. (d) Transcript levels of lipid metabolism genes in liver. (e) Transcript level of inflammation genes in gonadal WAT at E17.5. (N NC *n* = 16, H NC *n* = 6, N OD *n* = 9, H OD *n* = 6). *Gyg*, glycogenin;, *Gbe1*, glycogen branching enzyme 1; *G6pc*, Glucose‐6‐phosphatase; *Pfkl*, phosphofructokinase L; *Irs2*, insulin receptor substrate 2; *Pdk1*, pyruvate dehydrogenase kinase 1; *Srebpc1c*, sterol regulatory element binding protein 1c; *Acaca*, acetyl‐CoA carboxylase alpha; *Fasn*, fatty acid synthase; *Scd1*, stearoyl‐CoA desaturase‐1; *Ccl2*, chemokine (C‐C motif) ligand 2. All data are means ± SD. Two‐way ANOVA *p < 0.05, **p < 0.01, ***p < 0.001, ****p < 0.0001. Student’s *t*‐test #*p* < 0.05

The amount of glycogen in the maternal liver, skeletal muscle, and kidney at end‐gestation varied greatly from tissue to tissue, being the highest in the liver (Figure 5a), where the hypoxic intervention reduced the amount of glycogen by ~20% relative to normoxia with both diets, without reaching statistical significance (Figure 5a). In the skeletal muscle and kidney the amount of glycogen was only measured in the OD dams, where the hypoxic intervention showed a tendency for higher glycogen levels in both tissues (Figure 5a). The mRNA level of glycogen (*Gyg*) was upregulated by the OD in the liver but not in the skeletal muscle or kidney (Figure 5b). Hypoxia upregulated glycogen branching enzyme 1 (*Gbe1*) mRNA levels in the liver relative to normoxia in the mice receiving the NC diet, whereas these levels were downregulated by the OD diet relative to the NC in the liver in normoxia and hypoxia and in skeletal muscle in normoxia (Figure 5b). Glucose 6‐phosphatase (*G6pc*) mRNA levels in the liver were downregulated by the OD relative to NC in normoxia, but the downregulation in hypoxia did not reach statistical significance (Figure 5b). Most importantly, maternal hepatic *G6pc* mRNA levels correlated positively with embryo weight (*r* = 0.54, (95%CI [0.26; 0.74]), *p *= 0.0005).

The glycolysis rate limiting enzyme and HIF target gene phosphofructokinase L (*Pfkl*) mRNA was upregulated by hypoxia in the livers of the NC dams relative to the situation in normoxia, whereas the OD dams failed to increase the transcript level in hypoxia (Figure 5c). In skeletal muscle upregulation of the *Pfkl* mRNA level by hypoxia was seen with both diets, but this failed to reach statistical significance (Figure 5c). mRNA for another metabolic HIF target gene, the insulin sensitivity‐increasing insulin receptor substrate 2 (*Irs2*), was downregulated by OD as compared with NC in the liver under hypoxic conditions, whereas the downregulation did not reach statistical significance in normoxia (Figure 5c). Maternal hepatic *Irs2* mRNA levels correlated negatively with maternal glucose (*r* = –0.40, (95% CI [−0.64;−0.08]), *p *= 0.02) and insulin levels (*r* = −0.66, (95% CI [−0.82;−0.41]), *p* < 0.0001) and with the HOMA‐IR score (*r* = −0.66, (95% CI [−0.82;−0.40]), *p* < 0.0001). In the gonadal WAT the OD led to a significant decrease in the mRNA levels of *Pfkl* and the key downregulator of OXPHOS, pyruvate dehydrogenase kinase 1 (*Pdk1*), relative to NC in normoxia, suggesting compromised energy metabolism in the adipose tissue (Figure 5c). No significant downregulation was seen in the hypoxia OD‐fed dams (Figure 5c).

### 3.6 Diet‐induced IR altered the transcription of maternal hepatic lipid metabolism genes and increased the inflammatory response in the WAT, the latter being counteracted by hypoxia

In line with the increased serum insulin levels, the OD increased the transcript levels of sterol regulatory element binding protein 1c (*Srebp1c*) in the liver under conditions of both normoxia and hypoxia (Figures 4b, 5d). The OD also upregulated transcript levels of acetyl‐CoA carboxylase alpha (*Acaca*) in normoxia but not in hypoxia, and the hypoxic intervention increased its mRNA levels in the NC mice (Figure 5d). Fatty acid synthase (*Fasn*) mRNA levels were downregulated by hypoxia in the OD‐fed dams (Figure 5d), and the OD also downregulated stearoyl‐CoA desaturase‐1 (*Scd1*) mRNA levels in normoxia and hypoxia, whereas the hypoxic intervention increased its transcription independent of diet (Figure 5d). Finally, the OD upregulated mRNA levels of the pro‐inflammatory chemokine (C‐C motif) ligand 2 (*Ccl2*) in maternal WAT under normoxia but not hypoxia, and these levels correlated positively with insulin levels (*r* = 0.47, (95% CI [0.14;0.70]), *p *= 0.0007) and HOMA‐IR scores (*r* = 0.57, (95% CI [0.28;0.77]), *p *= 0.0007) (Figures 4b,c, 5e).

### 3.7 Hypoxia increased placental necrosis in end‐pregnancy in the cases of diet‐induced IR

**FIGURE 6:**
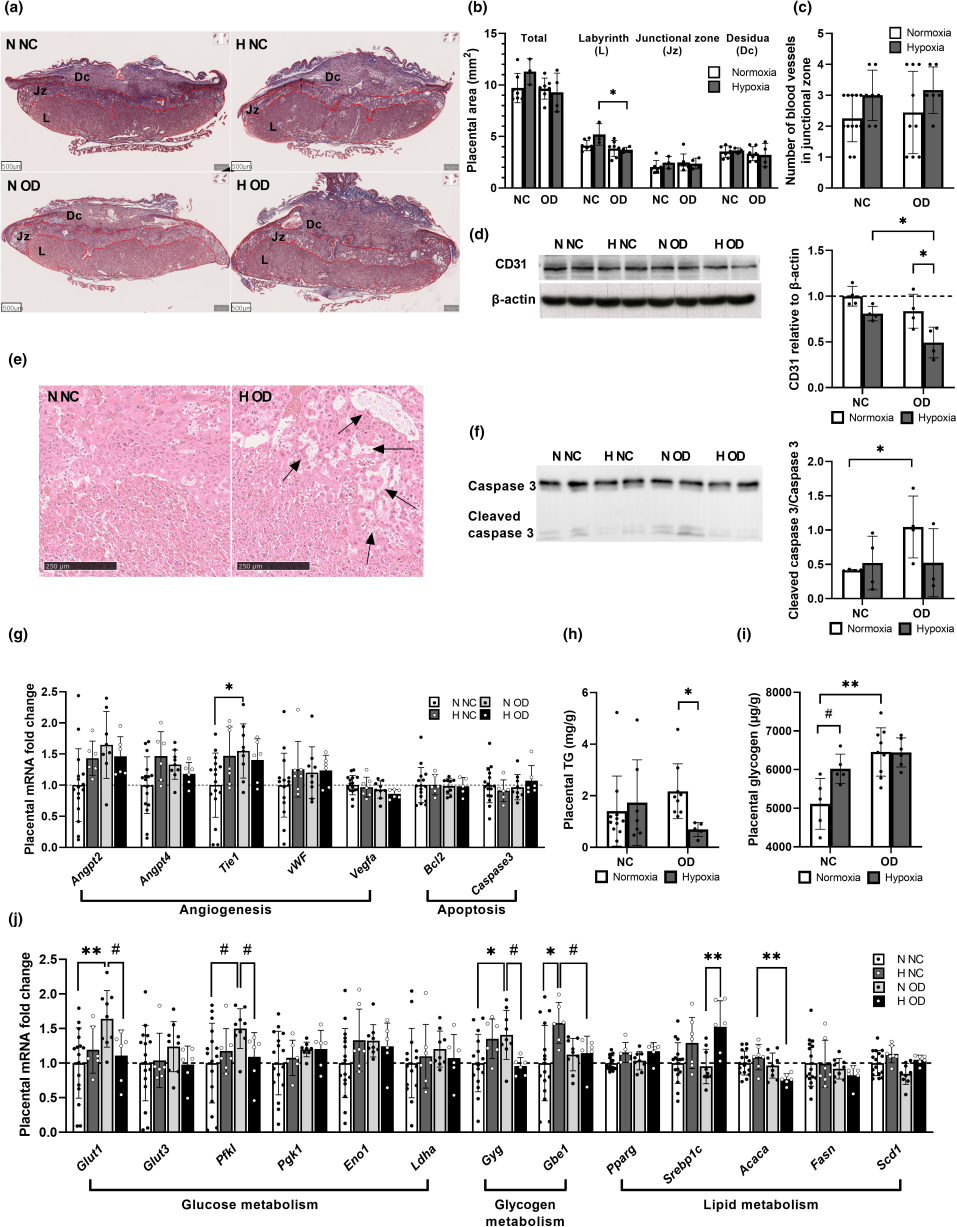
Hypoxia attenuated the placental outcome during end‐pregnancy. Mice were fed either normal chow (NC) or an obesogenic diet (OD) to induce insulin resistance. During gestation they were housed either in normoxic (N, O_2_ = 21%) or hypoxic (H, O_2_ = 15%) conditions. The parameters were measured at E17.5. (a) Overview of placental histology stained by Masson’s Trichrome, scale bars 500 µm. L = labyrinth zone, Jz = junctional zone, Dc = decidual layer. (b) Quantification of placental layer area (N NC *n* = 7, H NC *n* = 7, N OD *n* = 3, H OD *n* = 4). (c) Quantification of the number of blood vessel cross‐sections in the junctional zone (N NC *n* = 12, H NC *n* = 7, N OD *n* = 9, H OD *n* = 6). (d) Western blot and quantification of CD31 in placenta (N NC *n* = 4, H NC *n* = 4, N OD *n* = 4, H OD *n* = 4). (e) A close‐up of the junctional zone histology showing necrotic areas with arrows, scale bar 250 µm. (f) Western blot and quantification of cleaved caspase 3 in placenta (N NC *n* = 4, H NC *n* = 3, N OD *n* = 4, H OD *n* = 4). (g) Placental mRNA levels of angiogenesis and apoptosis related genes (N NC *n* = 16, H NC *n* = 7, N OD *n* = 9, H OD *n* = 6). (h) Placental triglyceride (TG) content N NC *n* = 11, H NC *n* = 9, N OD *n* = 7, H OD *n* = 5). (i) Placental glycogen at E17.5 (N NC *n* = 5, H NC *n* = 5, N OD =9, H OD *n* = 6). (j) Placental mRNA levels of glucose, glycogen and lipid metabolism related genes (N NC *n* = 16, H NC *n* = 7, N OD *n* = 9, H OD *n* = 6). *Angpt2*, angiopoietin 2; *Angpt4*, angiopoietin 4; *Tie1*, tyrosine kinase with immunoglobulin‐like and EGF‐like domains 1; *vWF*, vonWillebrand factor; *Vegfa*, vascular endothelial growth factor a; *Bcl2*, b‐cell lymphoma 2; *Glut1*, glucose transporter 1; *Glut3*, glucose transporter 3; *Pfkl*, phosphofructokinase L; *Pgk1*, phosphoglycerate kinase 1; *Eno1*, enolase 1; *Ldha*, lactate dehydrogenase a; *Gyg*, glycogenin; *Gbe1*, glycogen branching enzyme 1; *Pparg*, peroxisome proliferator‐activated receptor gamma; *Srebpc1c*, sterol regulatory element binding protein 1c; *Acaca*, acetyl‐CoA carboxylase alpha; *Fasn*, fatty acid synthase; *Scd1*, stearoyl‐CoA desaturase‐1. All data are means ± SD. Two‐way ANOVA *p < 0.05, **p < 0.01, ***p < 0.001, ****p < 0.0001. Student’s *t*‐test #*p *< 0.05, ##*p *< 0.01

No difference according to diet or hypoxic intervention was seen in the placental area as a whole, its junctional zone or its decidual area, whereas hypoxia reduced the labyrinth area in the OD cases but not the NC ones (Figure 6a,b, S2). No significant difference in the number of blood vessels in the junctional zone was detected between the diets, but the hypoxic intervention showed a tendency to lead to a higher number in the case of both diets (Figure 6c). Western blotting indicated lower protein levels of CD31 in the hypoxic OD placentas compared to the hypoxic NC and normoxic OD cases, this being indicative of a decrease in overall placental vasculature (Figure 6d). Interestingly, the placental protein levels of CD31 correlated positively with the number of embryos (*r* = 0.65, (95% CI [0.22;0.86]), *p *= 0.007) and embryo weight (*r* = 0.74, (95% CI [0.38;0.90]), *p *= 0.0011). The placentas of the hypoxia OD‐fed dams presented with large areas of cell death in the junctional zone and labyrinth, compromising normal functioning (Figure 6e arrows). Moreover, the protein levels of cleaved caspase three were increased by the OD as compared with NC in the normoxic placentas but not in the hypoxic ones (Figure 6f). These data suggest less apoptosis in the hypoxic than in the normoxic OD placentas, so that the cell death detected in the histological sections in the former would be necrotic rather than apoptotic (Figure 6e,f). Next, the transcript levels of apoptosis and angiogenesis related genes were examined. Apart from upregulation of tyrosine kinase with immunoglobulin‐like and EGF‐like domains 1 (*Tie1*) by OD in normoxia, no change in angiogenesis related genes were seen (Figure 6g). The apoptosis‐related b‐cell lymphoma 2 (*Bcl*‐*2*) and caspase 3 were likewise unchanged, in agreement with the observation that the regulation of apoptosis occurs mainly at the level of protein cleavage (Figure 6f,g).

### 3.8 Hypoxic intervention compromised placental energy metabolism in maternal diet‐induced IR

The amount of placental triglyceride was not altered by the OD in normoxia, whereas it was significantly less in the hypoxia OD‐fed dams (Figure 6h). By contrast, the OD increased the amount of glycogen in the placenta by ~18% compared with NC in normoxia, whereas no such additive effect was seen in hypoxia (Figure 6i).

Next, we examined placental mRNA levels to gain a deeper understanding of the molecular mechanisms behind the metabolic changes. The mRNA levels of glucose transporter 1 (*Glut1*), *Pfkl*, and *Gyg* were significantly upregulated by OD in normoxia, whereas the combination of OD and hypoxia significantly downregulated them all (Figure 6j). *Gbe1* mRNA was upregulated by hypoxia in the NC individuals but not in OD (Figure 6j). In response to decreased triglyceride levels in the OD hypoxia placenta, mRNA levels of *Srebpc1c* were upregulated by hypoxia in the OD‐fed dams while downregulation of *Acaca* was seen by the OD‐diet in hypoxia (Figure 6j). Altogether, these results suggest compromised energy metabolism in the hypoxia OD placentas at the end‐pregnancy stage.

## 4 DISCUSSION

As well as becoming a more common challenge for maternal and fetal health during pregnancy, GDM also increases the long‐term risks of cardiometabolic diseases for the mother and offspring (Damm et al., 2016; Vääräsmäki, 2016; Zhang et al., 2016; Zhu & Zhang, 2016). We evaluated here activation of the hypoxia response by environmental manipulation as a novel means of alleviating the detrimental effects of diet‐induced IR on maternal metabolism and the fetal outcome.

Our data show that feeding an OD to non‐pregnant female mice increased their fasting glucose and insulin levels and HOMA‐IR score and when continued during pregnancy led to a significantly more obesogenic and adipogenic maternal outcome with higher IR and poorer glucose tolerance but did not increase fasting glucose levels any further during pregnancy relative to NC. This model, although not fully phenocopying GDM where inappropriate modulation of the maternal metabolic environment by placental hormones is exacerbated in obese or IR individuals, has been used to study IR during pregnancy in mice (Fernandez‐Twinn et al., 2017). The OD increased the expression of the inflammatory *Ccl2* mRNA in WAT as compared with NC, and this had a significant positive correlation with both maternal insulin levels and HOMA‐IR scores, as befits the key role of adipose tissue inflammation in the formation of IR (Fuentes et al., 2013; Harford et al., 2011). Maternal serum cholesterol levels were not altered in the cases of diet‐induced IR compared with NC, whereas it led to lowered serum triglyceride levels and increased serum ketoacid levels. Regarding the fetal outcome, the diet‐induced IR did not significantly alter the number of embryos, but it did reduce the embryo weight. We suggest as one of the contributing factors to this difference is the reduced hepatic gluconeogenetic *G6pc* mRNA levels detected on the OD.

Our data show that hypoxic exposure is a potent means of alleviating maternal metabolism regardless of the diet pursued. The effects of our hypoxic intervention on NC were in line with earlier data, most notably the decrease in maternal weight gain during gestation and improved glucose tolerance (Määttä et al., 2018). Interestingly, when the dams with diet‐induced IR were placed in environmental hypoxia for the duration of their pregnancy the OD‐mediated increases in maternal weight gain and body weight, WAT weight and adipocyte size and the triglyceride levels in the liver and WAT as compared with the normoxic controls were revoked and many of these measures were reduced to the levels seen in dams receiving NC under conditions of normoxia. The hypoxic intervention also reduced the induction of *Ccl2* mRNA levels in the gonadal WAT of the OD‐fed dams, reflecting reduced WAT inflammation and improved insulin sensitivity. The combination of hypoxia and OD increased fasting serum cholesterol levels, principally HDL, compared with either normoxia and OD or hypoxia and NC, however, these levels being <3 mmol/l. Together with the reductions in tissue triglyceride levels, this may indicate maternal lipodystrophy as an outcome of the combination of diet‐induced IR and hypoxia. Hypoxia also reduced maternal liver weight during late gestation compared with normoxia in the dams with diet‐induced IR. As maternal liver weight is positively associated with the number of embryos (Milona et al., 2010), the failure to increase it in hypoxia to the similar extent as in normoxia during the period from E9.5 to E17.5 is probably associated with the detrimental outcome of an OD on fetal development. It has been shown that both maternal hypoxia and diet‐induced IR can have differential effects on placenta and offspring dependent on sex (Aiken & Ozanne, 2013; Cuffe et al., 2014; Gallou‐Kabani et al., 2010; Hellgren et al., 2021; Mao et al., 2010; Nivoit et al., 2009). Unfortunately, the information of the offspring sex was not collected here and therefore further analyses on this aspect could not be carried out.

Although gestational interventions, especially therapeutic ones, should be evaluated very carefully and may not prove desirable, the risks involved with GDM and its increasing prevalence do call for novel therapies. It has been shown earlier in a diet‐induced IR model that an exercise‐mediated improvement in maternal insulin sensitivity rescued the insulin sensitivity of her male offspring, indicating that hyperinsulinemia is a key programming factor, and therefore an important interventional target, during obese pregnancy (Fernandez‐Twinn et al., 2017). Moreover, in a genetic GDM model involving db/+ mice, hypoxia/reoxygenation treatment (1 h 6% O_2_ followed by reoxygenation at 21% O_2_ once a day), markedly ameliorated β‐cell insufficiency and glucose intolerance, suppressed oxidative stress and stimulated antioxidant enzymes, leading to improved reproductive outcomes (Hou et al., 2021). The sustained moderate environmental hypoxia (15% O_2_) used in the present work significantly improved the glucose tolerance and HOMA‐IR scores of the dams with diet‐induced IR compared with normoxia, even maintaining the levels seen in the NC controls, which could indicate beneficial effects on maternal metabolism. On the other hand, the combination of hypoxia and diet‐induced IR was highly detrimental to placental and fetal outcomes, reducing the number of embryos and their weight, the latter even more than the OD by itself. Therefore, it must be said that even this level of moderate environmental hypoxia, at least during the whole length of gestation, is not safe. Whether a safe and beneficial outcome could be achieved via the subjection of IR dams to environmental hypoxia e.g. only during end‐gestation remains to be studied. However, the data available to date indicate that especially severe or extreme hypoxia at end‐gestation reduces the fetal weight during this intense period of growth and may therefore not be desirable (Siragher & Sferruzzi‐Perri, 2021). After all, hypoxia has a very high potential for modulating metabolism, as confirmed here, the activation of a hypoxia response may be beneficial for obese women before pregnancy or for GDM mothers after delivery. Potentially, the offspring of GDM mothers, who are at risk for cardiometabolic diseases later in life, could also benefit from activation of the hypoxia response. Interestingly, studies assessing maternal early pregnancy Hb levels and GDM risk have indicated that among the normal variation of Hb levels, lower Hb levels associate with a reduced risk of GDM and some other cardiometabolic dysfunctions without having detrimental effects on fetus or placenta (Abeysena et al., 2010; Sissala et al., 2022). As Hb is the main carrier of oxygen, the lower Hb levels associate with lower tissue oxygenation which, when compared to individuals with higher Hb levels, may indicate hypoxia and have been shown to associate with upregulation of HIF target genes (Auvinen et al., 2021).

Interestingly, the diet‐induced IR lowered maternal Hb levels in association with heightened serum EPO levels, which is suggestive of an attempt to compensate for the decline in Hb levels by upregulation of erythropoiesis. Significantly lower serum EPO levels, associated with higher Hb levels, were also seen here in hypoxia relative to normoxia regardless of diet. On the other hand, the mRNA levels of hepatic hepcidin were downregulated in the diet‐induced IR mice relative to the controls, suggesting that the decline in Hb levels was not due to reduced recycling of iron from tissue stores or reduced intestinal intake, features which are typically associated with upregulation of hepcidin levels and inflammation. Apart from erythropoietic effects, EPO has regulatory effects upon energy homeostasis, impaired signaling in non‐erythropoietic tissues leading to obesity and IR, and increased EPO levels associated with pre‐eclampsia towards the end of pregnancy (Dey et al., 2020; Gusar et al., 2020; Reinhardt et al., 2016; Teng et al., 2011).

The diet‐induced IR increased placental apoptosis in normoxia but not in hypoxia, upregulated mRNA levels of the glucose metabolism genes *Glut1*, *Pfkl* and *Gyg* and increased the amount of placental glycogen during end‐pregnancy. In maternal liver hypoxia downregulated glycogen levels, however this did not reach statistical significance. Glycogen is stored in a specific cell type of the mouse placenta (glycogen trophoblast cells). These cells increase in number and glycogen content until around E16 when they start to release more glycogen than is stored while they also migrate out of the junctional zone. Although higher placental glycogen levels could potentially be indicative of a deficiency in the use of glycogen to support fetal growth, since macrosomia was not detected in the present diet‐induced IR offspring in normoxia, the phenomenon is likely more complex and could mean changes to not only in glycogen content, but cell lineage or cell migration (Tunster et al., 2020). The combination of hypoxia and NC did not alter the placental parameters measured here, whereas the most detrimental effects of the combination of hypoxic exposure and diet‐induced IR were detected in the placenta. In particular, the decline in placental protein levels of CD31, indicative of a decline in the quantity of endothelial cells, and evidently associated with the lower number of embryos and the decline in their weight, appeared to be a key mediator of the detrimental fetal outcome. This is in agreement with data from *Hif*‐*p4h*‐*2* ^−/−^ placentas with elevated HIF levels, where large spaces not lined with endothelial cells were detected and the placental defects also included significantly reduced labyrinthine branching morphogenesis, widespread penetration of the labyrinth by spongiotrophoblasts and an abnormal distribution of trophoblast giant cells, together resulting in developmental lethality between E12.5 and E14.5 (Takeda et al., 2006). In case of maternal inhalation hypoxia, the effect on placental function is dependent on timing, duration and intensity of the intervention (Siragher & Sferruzzi‐Perri, 2021). The structure of placental vasculature, especially in the junctional zone, is detrimentally altered by intense hypoxia of ≤12% O_2_. Interhemal membrane thickness is increased (Higgins et al., 2016) and blood space area, chorionic plate artery number and diameter are decreased (Cuffe et al., 2014; Ganguly et al., 2019; HvizdoŠová‐KleŠčová et al., 2013; Thompson et al., 2016) leading to negative impacts on fetal development and viability. In addition, the blood flow to placenta and perfusion are compromised by severe hypoxia at late gestation (Thaete et al., 2004; Tomlinson et al., 2010). Also, high fat diet‐feeding caused IR has been shown to lead to placental dysfunction in E18.5 dams via elevation of hypoxic stress markers in placenta (Li et al., 2013).

As a continuous oxygen supply is vital for fetal growth, the reduced uterine artery diameter/blood flow detected in high altitude hypoxic pregnancies has been considered a central defect leading to the observed growth retardation (Jensen & Moore, 1997; Moore et al., 2011; Zamudio et al., 1995). Although uterine blood flow was not measured here, the data from the hypoxia IR placentas indicated defects in vascularization associated with smaller fetal weight. Although the HIF response also mediates angiogenic effects, no signs of its upregulation were detected in the placentas of the NC or OD dams under hypoxic conditions. This highlights the fact that environmental hypoxia is not equal to activation of the HIF response alone. Indeed, genetic studies have associated SNPs in *PRKAA1*, coding for the catalytic subunit of AMPK, with the preservation of a normal uterine blood flow and fetal growth at high altitudes in Andean subjects (Bigham et al., 2014). Mice treated with an AMPK activator at mid‐gestation and then exposed to hypoxia until term had increased uterine artery diameters, greater uterine artery blood flow and only half of the altitude‐associated reduction in fetal growth found in their hypoxia‐exposed, vehicle‐treated counterparts (Lane et al., 2020). Interestingly, treatment of dams with diet‐induced obesity 1 week before mating and throughout pregnancy with metformin, which mode of action is AMPK activation, improved maternal metabolic health and reduced uterine artery compliance but did not correct placental structure or fetal growth restriction (Hufnagel et al., 2021). Taken together, our data suggest that activation of the hypoxia response could be a means for reducing maternal IR but is incapable of supporting fetal growth and survival, which are probably highly dependent on other factors such as placental functions and blood flow.

## CONFLICT OF INTEREST

The authors declared no conflict of interest.

## AUTHOR CONTRIBUTIONS

Niina Sissala: conceptualization, investigation, formal analysis, writing the original draft, visualization. Elisa Myllymäki: investigation, formal analysis, visualization. Florian Mohr: investigation, formal analysis. Riikka Halmetoja: investigation. Paula Kuvaja: investigation, formal analysis. Elitsa Y. Dimova: conceptualization, investigation, supervision. Peppi Koivunen: conceptualization, investigation, writing the original draft, supervision, project administration, funding acquisition.

## Supporting information



Supplementary Material
