# Supplementary material for: Hypoxia ameliorates maternal diet‐induced insulin resistance during pregnancy while having a detrimental effect on the placenta
Source: Physiol Rep. 2022 May 10;10(9):e15302. doi: 10.14814/phy2.15302 (PMC9088222; doi:10.14814/phy2.15302)
Supplement: Supplementary file 1 — Supplementary Material [file PHY2-10-e15302-s001.docx]

**Supplementary material**

**Hypoxia ameliorates maternal diet-induced insulin resistance during pregnancy while having a detrimental effect on the placenta**

Niina Sissala^1^, Elisa Myllymäki^1^, Florian Mohr^1^, Riikka Halmetoja^1^, Paula Kuvaja^2^, Elitsa Y. Dimova^1^ and Peppi Koivunen^1^*

^1^Biocenter Oulu and Faculty of Biochemistry and Molecular Medicine, Oulu Center for Cell-Matrix Research, University of Oulu, 90014 Oulu, Finland. 90014 Oulu, Finland.

^2^Finnish Institute for Health and Welfare, Oulu, Finland

*To whom correspondence should be addressed: Peppi Koivunen, MD, PhD, Faculty of Biochemistry and Molecular Medicine, University of Oulu, P.O. Box 5400, FIN-90014 University of Oulu, Oulu, Finland, Email: [peppi.koivunen@oulu.fi](mailto:peppi.koivunen@oulu.fi),Tel. +358294485822.

**Key words:** Gestational diabetes, glucose tolerance, hypoxia

**Running title:** Hypoxia and insulin resistance in pregnancy


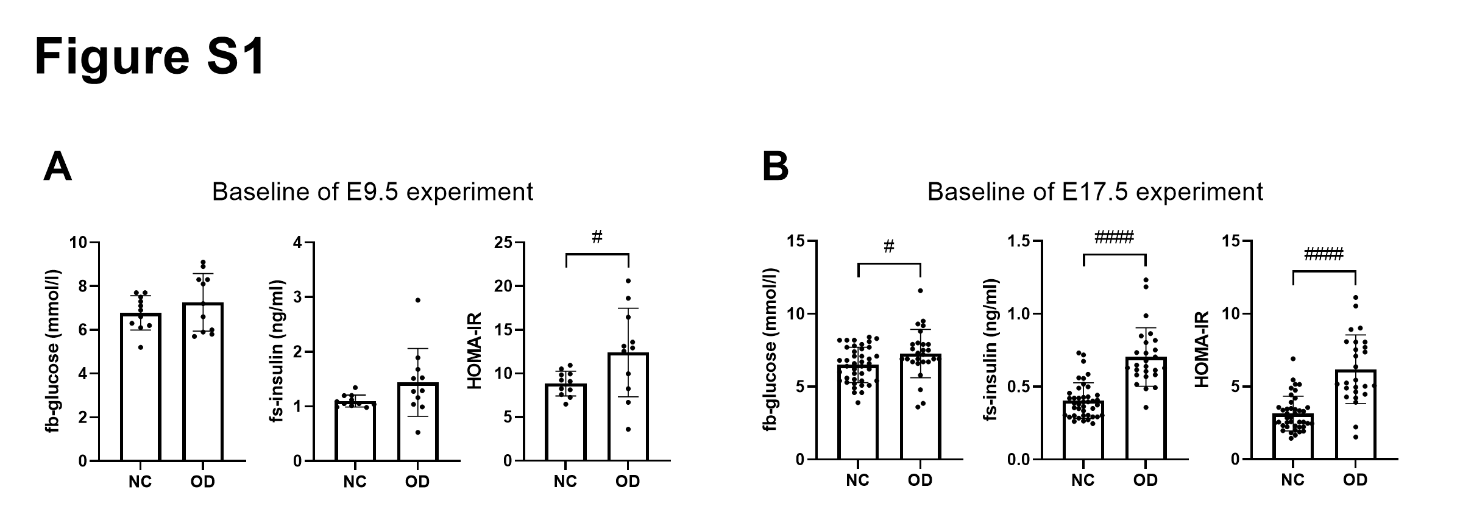


**Figure S1. High energy diet induced insulin resistance prior pregnancy.** Mice were fed either normal chow (NC) or an obesogenic diet (OD) to induce insulin resistance. A, fb-glucose, fs-insulin and HOMA-IR of non-pregnant females after 3.5 weeks on the diet (baseline), before hypoxic intervention in E9.5 experiment (NC n=11, OD n=11) and B, in E17.5 experiment (NC n=41, OD n=27).


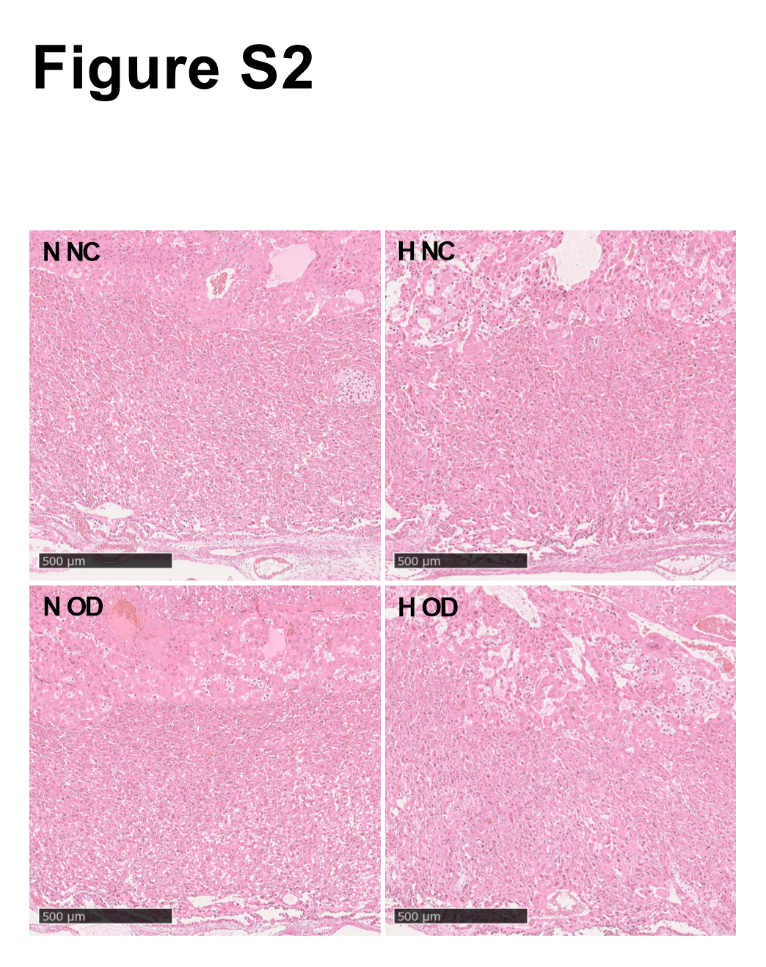


**Figure S2. Overview of placental histology at E17.5.** Stained by hematoxylin-eosin, scale bars 500µm. Mice were fed either normal chow (NC) or an obesogenic diet (OD) to induce insulin resistance. During gestation they were housed either in normoxic (N, O_2_=21%) or hypoxic (H, O_2_=15%) conditions.
